# A clustering method to identify who benefits most from the treatment group in clinical trials

**DOI:** 10.1080/21642850.2014.924857

**Published:** 2014-07-10

**Authors:** Beom S. Lee, Pranab K. Sen, Nan S. Park, Roger A. Boothroyd, Roger H. Peters, David A. Chiriboga

**Affiliations:** ^a^ Department of Mental Health Law & Policy, Louis de la Parte Florida Mental Health Institute, University of South Florida, Tampa, FL, USA; ^b^ Department of Biostatistics, University of North Carolina, Chapel Hill, NC, USA; ^c^ Department of Statistics and Operations Research, University of North Carolina, Chapel Hill, NC, USA; ^d^ School of Social Work, University of South Florida, Tampa, FL, USA; ^e^ Department of Child & Family Studies, Louis de la Parte Florida Mental Health Institute, University of South Florida, Tampa, FL, USA

**Keywords:** randomized controlled trial, treatment effectiveness, cluster analysis, substance abuse, motivational enhancement therapy

## Abstract

In randomized controlled trials (RCTs), the most compelling need is to determine whether the treatment condition was more effective than control. However, it is generally recognized that not all participants in the treatment group of most clinical trials benefit equally. While subgroup analyses are often used to compare treatment effectiveness across pre-determined subgroups categorized by patient characteristics, methods to empirically identify naturally occurring clusters of persons who benefit most from the treatment group have rarely been implemented. This article provides a modeling framework to accomplish this important task. Utilizing information about individuals from the treatment group who had *poor* outcomes, the present study proposes an a priori clustering strategy that classifies the individuals with initially *good* outcomes in the treatment group into: (a) group GE (good outcome, effective), the latent subgroup of individuals for whom the treatment is likely to be effective and (b) group GI (good outcome, ineffective), the latent subgroup of individuals for whom the treatment is not likely to be effective. The method is illustrated through a re-analysis of a publically available data set from the National Institute on Drug Abuse. The RCT examines the effectiveness of motivational enhancement therapy from 461 outpatients with substance abuse problems. The proposed method identified latent subgroups GE and GI, and the comparison between the two groups revealed several significantly different and informative characteristics even though both subgroups had *good* outcomes during the immediate post-therapy period. As a diagnostic means utilizing out-of-sample forecasting performance, the present study compared the relapse rates during the long-term follow-up period for the two subgroups. As expected, group GI, composed of individuals for whom the treatment was hypothesized to be ineffective, had a significantly higher relapse rate than group GE (63% vs. 27%; *χ* ^2^ = 9.99, *p*-value = .002).

## 1. Introduction

The randomized controlled trial (RCT) is considered as a gold standard for evidence-based practice in a variety of clinical fields including medicine, public health, pharmacy, and behavioral sciences. The conventional way to assess treatment effectiveness in RCTs is a simple comparison between the treatment group and the control group in terms of the mean of an outcome variable or the proportion of individuals with successful outcomes. After this basic analysis is completed, the researchers frequently conduct subgroup analysis to examine whether the treatment effect is superior or inferior in pre-determined subgroups of individuals. Subgroup analyses generally divide participants according to such baseline characteristics as age, gender, or race/ethnicity and evaluate treatment effects across homogeneous subgroups. More detailed explanations regarding subgroup analysis, including how to conduct it, how to report the results, and the cautions to be used in implementation, have previously been appeared in the literature (Assmann, Pocock, Enos, & Kasten, 2000; Lagakos, 2006; Pocock, Assmann, Enos, & Kasten, 2002; Rothwell, 2005; Yusuf, Wittes, Probstfield, & Tyroler, 1991).

A series of articles published in *Science* (Browner, 2003; Cohen, 2003a, 2003b; Nowak, 1994a, 1994b; Stanley, Fischl, & Collier, 1994) have revealed the existence of considerable debate and concerns associated with traditional approaches for conducting subgroup analysis within RCTs. In response to a presentation summarizing the results of a large clinical trial of anti-HIV drugs at the ninth international AIDS conference in 1993, for example, Nowak (1994a) expressed serious concerns about the subgroup analysis that was conducted and argued that the approach exaggerated the treatment effects of certain groups of patients. The study investigators responded that the subgroup analysis was conducted in a traditional and valid manner (Stanley et al., 1994), but Nowak (1994b) further responded that the subgroup analysis was still a questionable approach, even if it was implemented appropriately. In 2003, VaxGen released the results of the first-ever clinical trial of an AIDS vaccine and demonstrated more favorable outcomes of the AIDS vaccine among particular racial/ethnic groups. Cohen (2003a, 2003b) subsequently provided a summary critique of the subgroup analyses used in the study of the VaxGen AIDS vaccine, and Browner (2003) also argued that the study misused subgroup analysis.

Although the importance of subgroup analysis for providing information about differential efficacy and for future research is beyond dispute, the literature thus suggests that the most commonly employed methodologies for such analyses introduce analytic challenges and can lead to overstated and misleading results (Wang, Lagakos, Ware, Hunter, & Drazen, 2007). One of the most frequently expressed concerns is that a series of subgroup analyses examining each of many patient characteristics increase the chance of spurious false-positive findings (Kent & Hayward, 2007). Also, conventional subgroup analyses with one-variable-at-a-time approach would easily fail to identify the subgroup that should be described simultaneously with multiple characteristics (Hayward, Kent, Vijan, & Hofer, 2006).

As a way to cope with the problem of the univariate approach, Hayward et al. (2006) and Kent and Hayward (2007) advocated multivariate risk-stratified subgroup analysis, which builds a risk score by combining multiple patient characteristics and compares subgroups based on the risk score along with the treatment effect. Although this approach has the advantage of increasing the statistical power of detecting treatment heterogeneity across subgroups, its limitations include: (a) it requires the independent development of risk-prediction tools prior to the particular study (Kent et al., 2002) and those tools should be adapted and validated for the specific RCTs and (b) unlike the conventional subgroup analysis, it has no ability to examine individual factors that directly modify the treatment effect (Hayward et al., 2006). As a multivariate strategy that reduces these limitations, the method proposed in this article assesses multiple variables simultaneously and it does not require any externally developed risk-prediction tools because it uses existing variables as they are rather than generating additional risk scores. Furthermore, by identifying treatment moderators or mediators, the proposed method can identify naturally occurring subgroups of patients who have different effect sizes (Kraemer, Frank, & Kupfer, 2006; Kraemer, Wilson, Fairburn, & Agras, 2002).

Unlike the other subgroup analyses mentioned previously, the proposed approach does not compare pre-determined subgroups. Instead, the heterogeneous latent subgroups are generated directly from analysis of clinical trial data. Specifically, the approach uses person-centered modeling to identify subgroups of persons for whom the treatment is effective or ineffective rather than using groups pre-defined according to single variables such as gender, race, age, or risk score. In contrast, the conventional subgroup analysis may decompose the population into men and women and then compare the two subgroups. Similarly, the multivariate risk-stratified subgroup analysis may compare the subgroup of high risk scores with the subgroup of low risk scores for the treatment effectiveness. Advantages of using a person-centered approach over a variable-centered approach include: (i) inconsistent findings across studies and spurious relationships among variables can be solved in part by classifying persons into naturally occurring subgroups; (ii) findings can be generalized to groups of people; and (iii) the person-centered approach is inclusive of subgroups that deviate from the means such as outliers (Everitt, Landau, Leese, & Stahl, 2011).

An excellent example of person-centered modeling approach is the work of Kalichman, Cain, Knetch, and Hill (2005), who used a two-stage multivariate cluster analysis and identified three distinct heterogeneous subgroups of sexual risk behavior changes among patients who received risk reduction counseling. Kalichman et al. (2005) then used the outcome variables of the clinical trial to identify multiple subgroups among all patients in the treatment group. The approach proposed in the present article, however, seeks to identify the subgroup of the persons who are likely to benefit most from the treatment among the persons who did improve. For the actual clustering algorithm, this method directly uses baseline characteristics along with the profiles of the persons who did not get improved. Because the proposed method focuses on the treatment effectiveness for latent subgroups rather than pre-determined subgroups, it will be called latent group effectiveness modeling (LGEM) throughout the article.

## 2. LGEM model

**Figure 1.**
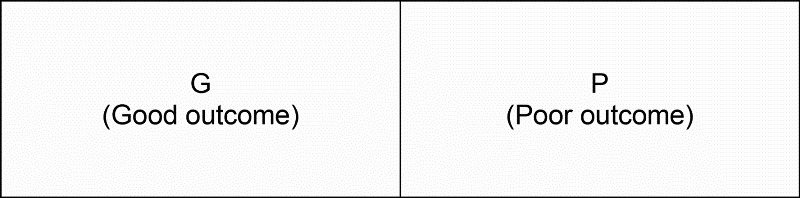
Decomposition of treatment group.

Consider the following scenario. In conventional analysis, after an RCT is completed, researchers may contrast pre-determined groups such as men and women, or younger vs. older. Alternatively, researchers may identify two subgroups of individuals in the treatment group, one (G) with relatively *good* outcomes and another (P) with relatively *poor* outcomes (see Figure 1). Researchers may compare these two observed groups, G and P, in order to identify the characteristics of the individuals who received benefits directly from the treatment. For example, if the proportion of female is significantly higher in group G than in group P, it might be concluded that the treatment is more effective for females. However, this intuitive method does not necessarily work to identify real beneficiaries from the treatment, as empirically shown in the results section.

**Figure 2.**
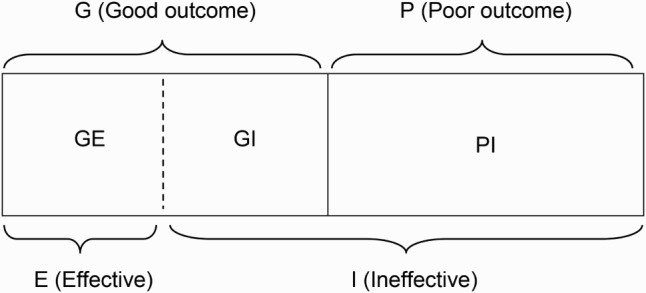
Further decomposition of treatment group.

As presented in Figure 2, the proposed LGEM approach addresses this problem from a different perspective. In addition to allowing the researcher to distinguish between individuals in the treatment group with good and poor outcomes, it allows the group G to be further broken down into two unobserved subgroups: a group GE (good outcome, effective) of the individuals who attained *good* outcomes probably because the treatment was *effective* for them and a group GI (good outcome, ineffective) of the individuals who initially attained *good* outcomes probably because of chance or some other reasons, but whose response to the treatment condition subsequently might have degraded. In short, the treatment for group GI may actually be *ineffective*. Because no direct treatment effectiveness can be found in group P, it can consist of only one type of individuals; group PI or the individuals for whom the treatment was *ineffective* under this framework. Once the decomposition of group G into the GE and GI subgroups has been established, this classification would now identify the characteristics of individuals who are more likely or less likely to receive actual benefits from the treatment or intervention; i.e. the classification into either group E of the individuals for whom the treatment is likely to be *effective* or group I of the individuals for whom the treatment is likely to be *ineffective*, as presented in the bottom of Figure 2.

To this point, the above-described approach might seem to be simply a matter of *post hoc* evaluations of how a previously effective outcome group breaks down into more and less effective groups. While such an analysis might suggest categorical variables that make a difference, what is proposed here is the development of predictive models. The practical problem is how to actually classify group G individuals into the GE and GI subgroups, a priori. The decomposition can be done using various techniques in cluster analysis. Cluster analysis encompasses a wide range of numerical methods that summarize data with a small number of groups or clusters and is often described as a technique for data reduction; that is, the clustering procedures generate groups of objects that resemble each other in the same cluster and that are different from the objects in other clusters (Everitt et al., 2011).

However, cluster analysis alone is not enough to accomplish the task of decomposition in the current scenario. What is proposed is that group PI in Figure 2 be hypothesized to consist of individuals who are unlikely to respond to treatment and that group G then be examined to identify individuals who actually share a profile of characteristics with group PI. Those who share a profile of characteristics with group PI are then classified as belonging – potentially – to a group whose positive response to treatment is likely to have occurred by chance and are labeled as group GI. Thus, LGEM incorporates information about group PI characteristics in order to identify members of the G group who may in fact constitute a GI subgroup. Because group PI is closer to group GI than group GE on a computed measure of proximity or distance based on observed participant characteristics, sustainable outcomes can be questioned. As such, groups PI and GI will make one cluster together as LGEM is implemented and the procedure will separate group GE into another cluster. This new modeling framework is the central idea of LGEM and allows researchers to test hypotheses about the characteristics of people who manifest a sustained positive response to treatment.

LGEM can be further extended to extract additional information from RCTs by utilizing the data collected from the control group. Group PI is used as the reference group for decomposition in the basic form of LGEM as previously described. However, there exists another observable reference group that can be used in addition to group PI in the process of classifying group G into groups GE and GI. Conceptually, the individuals in group GI can be viewed in the same manner as the individuals who were assigned to the control group but attained good outcomes given that their good outcomes are the result of the factors other than the treatment. This subgroup in the control group will be called group CG (control group, good outcome), and it can be readily used as another reference for decomposition of group G into groups GE and GI in the sense that group CG is observable and likely to be closer to group GI compared to group GE because groups CG and GI get improved without the treatment effect. This additional classification using group CG as the reference can complement or confirm the basic classification using group PI as the reference. If the analysis utilized the basic classification by group PI only, though, it could be applied not only to supplement analyses of RCT data but also to any observational studies of treatments or interventions because there is no need of control group data. This is another significant advantage of using LGEM in subgroup analyses.

In the actual classification to be described next, the present study used a classical clustering algorithm called the partitioning around medoids (PAM) algorithm (Kaufman & Rousseeuw, 2008). A medoid is the object with the minimum absolute distance to the other members of the cluster (Everitt et al., 2011), so it is the most representative member in the group. The *k*-means algorithm is more frequently used due to its computational simplicity. However, the *k*-means algorithm is more sensitive to outliers and noise, and in principle it is not suitable for categorical data (Theodoridis & Koutroumbas, 2008). The PAM algorithm is more robust to outliers and noise, and it can appropriately deal with categorical data, which are very common in RCTs. For the distance measure used in the clustering algorithm, the dissimilarity measure of Gower (1971) was used because it can handle both continuous and categorical baseline characteristics simultaneously in a single model. The conventional algorithm of PAM was modified to address LGEM in the present study and the basic algorithm of LGEM using group PI as the reference is described as follows.

*Step 1*: Find the medoids of G and P using multiple baseline characteristics, then set them to be the medoids of E and I, respectively, as in Figure 2.
*Step 2*: Relocate the individuals in group G to either group E or I using the closer medoid to each individual correspondingly. Notice that the individuals in group PI will never be relocated because they are known to belong to group I.
*Step 3*: Find new medoids of newly assembled E and I.
*Step 4*: Repeat steps 2 and 3 until there is no change in the medoids.

For the form of LGEM using group CG as the reference, the algorithm simply replaces P or PI with CG.

## 3. Empirical illustration

### 3.1. Example

To provide an applied illustration of the LGEM method, data provided by the National Institute on Drug Abuse (NIDA) through the National Drug Abuse Treatment Clinical Trials Network (CTN) were acquired. The present study selected the data from a multisite RCT, whose study number is NIDA-CTN-0004. The reason for selecting this RCT is that it easily comprehensible for laypersons, and included the two follow-up data points that are necessary to conduct diagnostics of the LGEM method. If the goal of the present study did not involve diagnostics, only one follow-up period would be sufficient to implement the LGEM method. In addition, when implementing the LGEM method, there is no limitation in terms of the time point or the length of the follow-up period as long as the criteria for determining better and worse outcomes are clear.

The data are publically available at www.ctndatashare.org. The RCT examines the effectiveness of motivational enhancement therapy (MET; Miller, Zweben, DiClemente, & Rychtarik, 1992) compared to counseling as usual (CAU) from 461 outpatients with substance abuse problems. According to Ball et al. (2007), primary outcomes article, the study implemented three-session interventions with either MET or CAU and resulted in reductions in self-reported days per week of primary substance use during the four-week therapy period. During the subsequent 12-week follow-up period, however, only MET participants sustained reductions while CAU participants increased substance use to baseline levels. MET did not demonstrate significantly better treatment effects compared to CAU in terms of either the retention or urine drug test outcomes. Conducting separate evaluations of the treatment effectiveness for two subgroups of primary alcohol users and primary drug users, Ball et al. (2007) managed to find that MET was associated with more sustained reductions than CAU among primary alcohol users only.

This re-analysis of the NIDA-CTN-0004 data was based on 289 individuals with no missing data. While the analysis could have been conducted with missing data using the dissimilarity measure of Gower (1971), the goal of this analysis was to demonstrate the mechanism of the LGEM procedure in a simple setting by excluding possible distortions that missingness may generate. Among participants included in this analysis, 142 individuals were in the MET group and 147 individuals were in the CAU group. Although the primary outcomes article (Ball et al., 2007) used continuous variables for the outcome measure of improvement, the present study used a dichotomous variable: whether the individual indicated no substance use from any interview or laboratory test during the post-therapy period (weeks 4–6). The reason of using a dichotomous variable is that the LGEM method requires a clear separation between good and poor outcomes as described in the model explanation section of this paper. The individuals with good outcomes (i.e. individuals reporting no substance use) during the post-therapy period were labeled as group G, and those with poor outcomes (i.e. individuals reporting substance use) were labeled as group P, as in Figures 1 and 2. Among the 142 individuals in the MET group, 83 individuals (58%; group G) appeared to have no substance use during the post-therapy period (weeks 4–6), while 59 individuals (42%; group P) appeared to have at least one time of substance use. Among the 147 individuals in the CAU group, 92 individuals (63%) had no substance use during the post-therapy period, while 55 individuals (37%) had at least one time of substance use. The CAU group therefore showed a slightly better outcome than the MET group (63% vs. 58%) during the immediate post-therapy period (weeks 4–6), but there was no statistically significant difference between two groups (*χ* ^2 ^= 0.52, *p*-value = .472).

LGEM was then implemented to decompose group G into group GE and group GI, using group PI characteristics as a reference. In contrast to the conventional subgroup analytic approach of comparing each variable separately, the goal here was to use the person-centered approach of LGEM in order to identify the latent subgroup of individuals for whom MET was more likely to be effective. Once the classification is completed using the LGEM approach, any descriptive statistical method can be used to investigate the characteristics of the identified individuals associated with a significantly greater likelihood of receiving treatment effects. As such, moderators or mediators of treatment effect can be identified as by-products of LGEM in the subsequent analyses. Although the method can utilize any selection of baseline variables, the present study selected variables by utilizing the diagnostic mechanism of LGEM in order to explore all the possible combinations of variables. The appendix explains how the procedure selected 11 baseline variables, presented in Table 1 or Table 2, out of 15 plausible candidate variables in this example. Using those 11 variables and group PI (poor outcome, ineffective) as the reference, the LGEM approach decomposed group G (*n *= 83) further into group GE (*n *= 51) and group GI (*n *= 32). Since this method uses a nonparametric clustering algorithm, there is no specific statistical inference available for the sample size and the sample size would not impact the effectiveness of this approach.

**Table 1.** Profiles of G (good outcome) and P (poor outcome).

Variable	G (*n *= 83)			P (*n *= 59)			*t*/*χ*^2^	*p*
	%	*M*	SD	%	*M*	SD		
Gender (female)	26.5			33.9			0.90	.341
Ethnicity							4.09	.252
Caucasian	37.3			49.2				
African American	43.4			39.0				
Hispanic American	10.8			10.2				
Other	8.4			1.7				
Age		35.99	10.66		33.43	11.02	−1.38	.169
Years of education		12.75	2.01		12.58	2.06	−0.49	.624
Primary drug of abuse							5.97	.309
Alcohol	34.9			44.1				
Cocaine	20.5			10.2				
Marijuana	13.3			22.0				
Methamphetamines	4.8			1.7				
Opioids	8.4			8.5				
Benzodiazepines	0.0			0.0				
Other	18.1			13.6				
With whom spend most of free time							0.92	.631
Alone	25.3			32.2				
Family	38.6			37.3				
Friends	36.1			30.5				
Do you stop using as a result of this treatment?							0.70	.872
I think I will still use	2.4			3.4				
I think I might stop	10.8			11.9				
I probably will stop	24.1			28.8				
I am sure I will stop	62.7			55.9				
No. of days paid for working		10.72	10.77		8.32	9.21	−1.43	.156
Been prescribed medication for any psychological/emotional problem	20.5			25.4			0.48	.487
No. of days experienced these psychological/emotional problems		3.66	7.76		5.92	10.16	1.43	.155
Serious depression	13.3			18.6			0.77	.382

Note: The last four variables are measures during the four-week therapy period.

**Table 2.** Profiles of GE (good outcome, efficient) and GI (good outcome, inefficient).

Variable	GE (*n *= 51)			GI (*n *= 32)			*t*/*χ*^2^	*p*
	%	*M*	SD	%	*M*	SD		
Gender (female)	21.6			34.4			1.66	.198
Ethnicity							30.66	.000
Caucasian	15.7			71.9				
African American	64.7			9.4				
Hispanic American	11.8			9.4				
Other	7.8			9.4				
Age		36.31	10.98		35.48	10.30	−0.35	.728
Years of education		12.43	1.72		13.25	2.33	1.72	.092
Primary drug of abuse							5.10	.404
Alcohol	29.4			43.8				
Cocaine	23.5			15.6				
Marijuana	17.6			6.3				
Methamphetamines	3.9			6.3				
Opioids	5.9			12.5				
Benzodiazepines	0.0			0.0				
Other	19.6			15.6				
With whom spend most of free time							7.78	.020
Alone	15.7			40.6				
Family	39.2			37.5				
Friends	45.1			21.9				
Do you stop using as a result of this treatment?							14.82	.002
I think I will still use	2.0			3.1				
I think I might stop	7.8			15.6				
I probably will stop	11.8			43.8				
I am sure I will stop	78.4			37.5				
No. of days paid for working		11.59	11.03		9.34	10.37	−0.94	.352
Been prescribed medication for any psychological/emotional problem	9.8			37.5			9.26	.002
No. of days experienced these psychological/emotional problems		1.45	4.54		7.19	10.25	2.99	.005
Serious depression	3.9			28.1			10.02	.002

Note: The last four variables are measures during the four-week therapy period.

### 3.2. Results

Before the comparison between groups GE and GI, the present study compared readily observable groups G and P in order to check whether the profiles of groups G and P are significantly different because if it is the case the description of group G would immediately provide the profiles of the individuals who are more likely to receive benefits from treatment. However, there were no significant differences between groups G and P for the 11 baseline variables used in the present study as shown in Table 1. On the other hand, once group G was decomposed into groups GE and GI, the comparison between groups GE and GI revealed several significantly different characteristics even if both of them had good outcomes during the immediate post-therapy period (weeks 4–6). Table 2 shows the profiles of groups GE and GI in terms of each baseline characteristic. The first significantly noticeable variable is ethnicity; group GE has a high proportion of the African American subjects (65%) whereas group GI has a high proportion of Caucasians (72%). Thus, the African Americans appear to receive more benefits from MET than the Caucasians. In terms of the variable pertaining to persons with whom most free time is spent, group GE has a lower rate of spending free time alone (16%) whereas group GI has a higher rate (41%), which supports the importance of social engagement as a moderator of MET effectiveness in the treatment of substance abuse. For the confidence to stop substance use, group GE expresses significantly stronger confidence than group GI, thus self-confidence appears to be an important factor in reducing substance use within the context of MET interventions. The patterns in the last three variables related to mental health problems in Table 2 suggest the individuals in group GE have fewer mental health problems than those in group GI; the result may suggest that mental health factors played an important role in mediating outcomes of MET treatment for substance abuse.

### 3.3. Diagnostics

**Figure 3.**
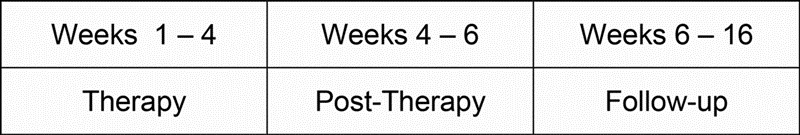
Three phases in the example.

It should be emphasized that the decomposition of group G into groups GE and GI in the example was made using the information only up to the post-therapy period (weeks 4–6) as shown in Figure 3. In order to confirm the hypothesis that group GI – due to its comparability with the characteristics of group PI – will deteriorate on further follow-up, the present study compared the relapse rates of groups GE and GI during the extended follow-up period (weeks 6–16). In other words, if LGEM clustering has discriminating power, group GI was expected to have a significantly higher relapse rate than group GE during the follow-up period (weeks 6–16) even though both groups had shown an improvement during the post-therapy period (weeks 4–6). Indeed, the relapse rates during the long-term follow-up period for groups GI and GE were 63% and 27%, respectively; this result supports the validity of results since the relapse rate of group GI was clearly higher than that of group GE (*χ* ^2^ = 9.99, *p*-value = .002).

## 4. Conclusion

It is generally recognized that not all participants in the treatment group of most clinical trials benefit equally. Not only is recognition of differential benefit helpful in understanding mechanisms of change, but also the identification of the treatment heterogeneity across latent subgroups of patients can prevent potential future harms to underrepresented subpopulation as described in Kraemer et al. (2002) and Kraemer et al. (2006). Researchers frequently conduct subgroup analysis that compares the treatment effectiveness between the treatment and control groups for each of baseline characteristics at a time. Although conventional subgroup analysis provides information about the heterogeneity of the treatment effectiveness across subgroups in terms of baseline characteristic variables, these analyses do not take account of the basic fact that each participant simultaneously has many characteristics, and that the mix of these characteristics will vary across participants. Similarly, while multivariate risk-stratified subgroup analyses have the advantage of using multiple variables simultaneously, the requirement of externally developed risk-prediction tools and the lack of ability to identify treatment moderators pose problems in interpretation. To fill the gap, a new strategy that makes maximum use of existing analytic approaches is proposed. The LGEM method is a modeling framework for which individuals are likely to have received the greatest or least treatment benefits in RCTs. Once the individuals for whom the treatment is likely to be most effective or ineffective are identified by LGEM, the profiles of those persons can be readily examined regarding important characteristics of interest such as the treatment moderators or mediators.

The additional advantages of LGEM can be summarized as follows. First, because LGEM uses multiple variables simultaneously in identifying the persons of interest, it properly eliminates problems related to confounding or interactions in subgroup analysis that have been noted by VanderWeele and Knol (2011). Second, because LGEM can be implemented after clinical trials are completed, without the need for a pre-specified design, it can be used to explore potentially new findings through simple re-analyses of existing data at no additional cost to the study and lends itself readily to secondary analyses. Third, LGEM will tremendously extend the range of the data that can be analyzed because it can be used not only for simple observational studies with any treatment or intervention but also for identification of the persons in the control group for whom the control condition is more likely to be effective. Finally, LGEM is a general mechanism which is not limited by the research topic; thus, it can be applied to any type of RCTs in a variety of areas including studies of cancer, HIV, mental illness as well as substance abuse. Finally, LGEM is a general mechanism that is not limited by the research topic, thus it can be applied to any type of RCTs, and in such diverse areas as studies of cancer, HIV, mental illness, and substance abuse. In all these areas, the method could, for example, lead to individualized treatment planning in health care settings by identifying persons who are most likely to benefit from specific treatments.

In the re-analysis of data from a study of treatment for substance abuse from the NIDA CTN, LGEM identified individuals for whom a new (MET) treatment was likely to be most effective. The resulting subject profiles were informative and the findings could not have been revealed through conventional methods. However, a caution should be noted regarding *post hoc* analysis after the implementation of LGEM; because LGEM uses multiple variables jointly, the interpretation of the results should recognize the multivariate context to correctly understand the findings. For example, non-significant variables from separate univariate comparisons might become jointly significant in the multivariate context. Also, the diagnostics method conducted in the present study might not be adequate to show that LGEM truly reveals what it claims. A more concrete evidence to support LGEM could be obtained from more rigorous empirical studies. For example, if LGEM is implemented to a different RCT with the same treatment used in the example of the present study and the resulting profiles of the subgroups are similar to the findings in this article, it will provide more sounding justification of using LGEM. Another caution should be made that LGEM does not provide the direct causality between the treatment and the benefits. Although the LGEM method can identify the latent subgroup of persons who potentially benefit most from the treatment group, it does not necessarily mean that the treatment itself is the direct cause of the benefit. More rigorous and systematic method should be devised to identify causality.

As a final point, the same approach taken with the treatment group could be undertaken with the control group, if the latter was exposed to an actual intervention. Here the interest would be in whether the control group actually has a positive impact on participants who share a particular constellation of characteristics. The LGEM method, in other words, has potential as a means of further exploring reasons why individuals respond to treatment conditions, regardless of which treatment arm they are exposed to.

## Appendix. Variable selection procedure (how to select variables to be used for LGEM)

If the decomposition of group G into group GE and group GI was completed accurately, then group GE would be expected to have a significantly lower rate of relapse than group GI during the follow-up period (weeks 6–16). Thus, the optimal procedure of variable selection becomes finding the combination of variables which gives the largest gap of the relapse rate between groups GE and GI. The basic form of LGEM using group PI as the reference was initially implemented for every possible combination of 15 variables, which include the 11 variables in Table 1 or Table 2 and four other variables: marital status (never married/divorced/legally married/separated), “Was this admission prompted or suggested by the criminal justice system?” (yes/no), “Have you been in controlled environments in past 30 days?” (yes/no), and “Are you on probation or parole?” (yes/no). This leads to a total of 32,752 possible combinations of variables. LGEM was then implemented independently for each of these combinations to decompose group G into groups GE and GI. Among those 32,752 different combinations of variables, there were 18 “best” combinations, which generated the largest gap in relapse rates between the GE and GI groups during the follow-up period. For the purpose of further narrowing down to the final best combination among those 18 combinations, the present study implemented another type of decomposition using group CG (control group, good outcome) as the reference group, which was described in the LGEM model section. This additional procedure reduced the number of best combinations to four, then the largest set of variables among those four combinations was selected to incorporate as much information as possible. The final 11 baseline characteristic variables were used for the example in the present study.
